# Protein supplementation and dietary behaviours of resistance trained men and women attending commercial gyms: a comparative study between the city centre and the suburbs of Palermo, Italy

**DOI:** 10.1186/1550-2783-11-30

**Published:** 2014-06-18

**Authors:** Antonino Bianco, Caterina Mammina, Ewan Thomas, Marianna Bellafiore, Giuseppe Battaglia, Tatiana Moro, Antonio Paoli, Antonio Palma

**Affiliations:** 1 Sport and Exercises Research Unit, University of Palermo, Via Eleonora Duse, 2-90146 Palermo, Italy; 2 “G. D’Alessandro” Department of Sciences for Health Promotion and Mother-Child Care, University of Palermo, Via del Vespro, 90129 Palermo, Italy; 3 Department of Biomedical Science, University of Padova, Via Manzolo 3, 35131 Padova, Italy

**Keywords:** Dietary behaviour, Protein project, Protein supplements, Questionnaire, Gym

## Abstract

**Background:**

It is anecdotally recognized that commercial gym users assume supplements in order to improve performance or health. However, dietary behaviours of people and athletes attending commercial gyms have been poorly studied. The exact amount and frequency of dietary supplements consumption are still needed to be investigated. The main purpose of this study is to understand the quantity and quality of food intake, as well as dietary supplementation in people attending commercial gyms. Secondly to compare the city centre and the suburbs of Palermo, Italy.

**Methods:**

A face-to-face questionnaire was administered to 561 subjects, 207 from the city centre (CC) and 354 from the suburbs (SB) of Palermo, Italy. Frequency of protein supplements use and association with dietary behaviours were investigated. Subsequently, the frequency distribution was used for demographic assessment.

**Results:**

Frequency of protein consumption was similar in both groups (30% for CC and 28.8% for SB). Males show greater consumption percentages than females (30.5% in males and 6.9% in females). Milk and chicken are the most frequently consumed foods. Data show that non-supplement users (NSU) consume significantly more snacks and bakery products than supplement users (SU) (P < 0.001). While, SU consume significantly higher quantities of vegetables, nuts, fresh fish, eggs and canned tuna (P < 0.001). SU consume less low protein food and higher protein foods than NSU. No differences were found between CC and SB.

**Conclusions:**

Protein consumption among commercial gym users is 30% for the CC and 28.8% for the SB. Significant differences were found between CC and SB females, underlining an interesting discrepancy, indicating to dietary supplement industries regarding regional implications. Subjects that use protein supplements also consume larger quantities of high protein food compared to NSU. NSU also eat higher proportions of unhealthy food compared to SU.

## Introduction

The use of supplements is a generally accepted and widespread practice for a variety of reasons. Health, physical appearance, performance and nutritional purposes are usually the main reasons inducing such consumption [1]. Active individuals use supplements to build muscle, gain strength or prevent future diseases and illnesses [2,3]. The typologies of supplements ingested are related to the age, gender, specific exercise programs and practiced sport [4-6]. Kaufman et al. found that older people were more likely to take multivitamin and mineral supplements, while younger people were more likely to take creatine [4]. Older adults are more likely to use supplements for site-specific health reasons (e.g., bone, heart, eye). Whereas, younger adults are more likely to use products with a short-term effect, either to enhance energy or boost immune function. It has also been reported by Bailey et al. that both men and women use supplements for very specific gender related reasons (e.g., heart and bone health, respectively) [7]. Furthermore, scientific researchers have shown that people have different opinions about the use of supplements [5,6,8-15] and the appropriate food to eat. As reported by Bianco et al. [16] and colleagues [5,6], proteins are the most widely ingested supplements in people attending commercial gyms. Moreover, there is an increased interest in what is considered “*proper*” nutrition [17-19]. However, gym users might follow dietary regimes that are less or more than optimal [20,21]. According to the nutrition transition model [22], the dietary patterns of a society become more diversified amidst urbanization and higher income levels. This dietary diversity is often associated with an increase in the proportion of fats and sweeteners [23]. Dietary behaviour is in fact a complex phenomenon; food-based approaches are regarded as the long-term strategy for improving nutrition. These require significant efforts and appropriate planning in order to include certain specific macronutrients or supplements in everyday’s diet [24]. Dieting or unhealthy eating practices, (such as eating foods deemed as “*bad*” by the dieter), may be associated with long-term weight gain [25].

The purpose of this investigation is to understand frequency of food intake of common foods and how this consumption varies between those who use dietary supplements and those who don’t. In addition we are interested in understanding the eventual differences between the city centre and the suburbs of Palermo in resistance trained men and women.

## Methods

### Participants

Permissions to conduct a survey were obtained from the managers of a representative number of twelve commercial gyms located in the suburbs of Palermo in 2013. We considered suburb gyms (SB) as being located on the outskirts of Palermo (Range from 20 km to 60 km). The gyms were identified by using a database of the CONI register (National Olympic Committee Register for Sport and Fitness Associations). Through this fitness database, a number of 1200 people (20% of the total number) (Age ranging between 13 and 68 years old 26 ± 9 yrs; Females 27 ± 9 yrs, Males 26 ± 9 for the CC and 29 ± 10 yrs, Females 31 ± 10, Males 29 ± 10 for the SB), were randomly selected as potential participants. Only fitness and gym attendees who were taking part in strength training courses (Gym, functional fitness, weightlifting, etc.…) have been selected. All gym and fitness users performing aerobic activities (such as aerobic, spinning, step, circuit training, endurance and cardiovascular programs, etc.…) were excluded. On the basis of these inclusion/exclusion criteria, a total of 354 participants were retained for the present investigation. These subjects were consequently compared with those from our previous study (207 participants), carried out in gyms located in Palermo City (CC) [16].

### Questionnaire procedure

In order to evaluate the frequency consumption of protein supplements amongst participants, dietary behaviours and other related information, the questionnaire method was adopted [13] (Additional file 1). The same questionnaire has been administered in commercial gyms of the suburbs of Palermo, Italy. Easy understandable definitions of the supplements were provided to the participants (Common and commercial names of products or substances included within the definition of supplement: product intended to supplement the diet that contains one or more dietary ingredients) [26]. Completion of the questionnaire implied the agreement of respective gym users to participate in the study. According to the Italian regulations, ethical approval was not required for this study. The same investigator using the face-to-face interview method during a period of six months administered the questionnaire.

### Food classification

Foods were categorized in accordance to their protein content in three categories: Low, medium and high. We considered low content foods with ≤ 10 g of proteins for 100 g of food, medium those with a protein content between 10 and 20 g every 100 g and finally, high content foods with 20-25 g or above accordingly. The protein content percentage of each food was retrieved from the INRAN database (Istituto Nazionale di Ricerca per gli Alimenti e la Nutrizione; Website: http://nut.entecra.it/646/tabelle_di_composizione_degli_alimenti.html).

### Data analysis

Data analysis was performed using the EpiInfo software version 7.0 (CDC, Atlanta, GA, US) and Statistica version 8.0 software for Windows (Tulsa, OK, US). The descriptive analysis was performed by calculating the means and standard deviations. Contingency tables were used to assess frequency distribution of protein consumption solely or stratified by gender, frequency of use and food. Differences were assessed by a two-way ANOVA test and a Bonferroni post-hoc test to compare replicate means by row. The associations between the categorical variables under examination were evaluated using contingency tables. Statistical significance was set at P values ≤ 0.05.

## Results

Power analysis showed a statistical power of 0.99 and an effect size of 0.6.

### Demographic results

561 questionnaires were analysed after the completion of the investigation. Gender stratification has showed 434 male and 137 female participants. The surveyed population was split between supplement users from the CC and the SB for comparison. The CC group comprised of 80 females and 127 male participants while SB group of 47 females and 307 male participants. The majority of the subjects were aged between 18 and 30 years of age.

**Table 1:** Percentage and type of dietary supplements used by all participants

	**Subjects**	
	**City centre (207)**	**Suburbs (354)**
**Supplements use**		
No	70%	71.2%
Yes	30%	28.8%
**Users of supplements by gender**		
Male	69.5%	93.1%
Female	30.5%	6.9%
**Frequency of use**		
1 time per wk	12.9%	1%
2 time per wk	8.1%	3.9%
3 time per wk	21.0%	32.3%
4 time per wk	17.7%	6.9%
5 time per wk	14.5%	49%
6 time per wk	1.6%	1%
7 time per wk	24.2%	5.9%

Palermo, Italy.

### Frequency distribution

**Figure 1:**
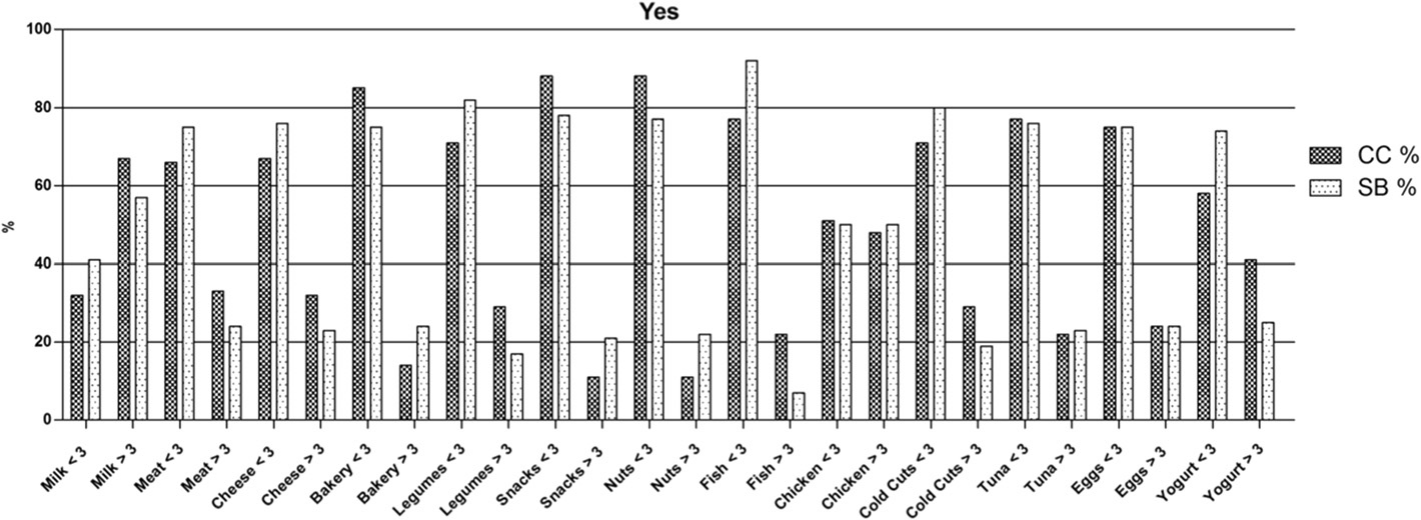
**Food intake percentage of people who use protein supplements.** The figure provides information about the frequency of consumption of gym users who use protein supplements and their weekly food intake divided in two categories: Greater than 3 times per week and 3 times or lower per week. The data are expressed as percentage.

**Figure 2:**
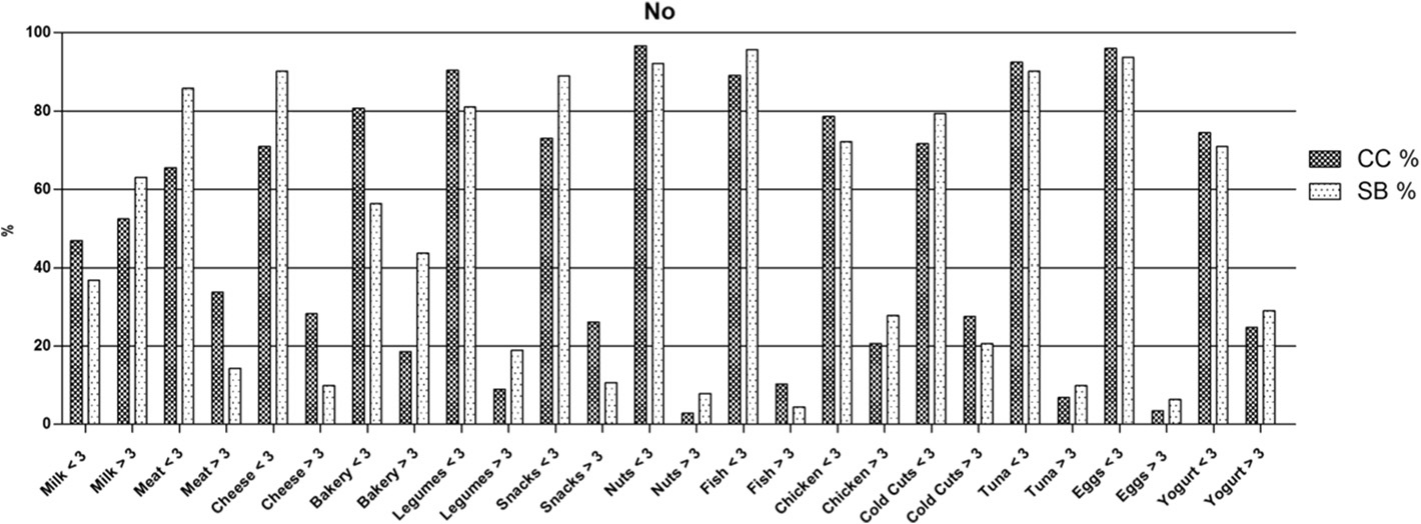
**Food intake percentage of people who don’t use protein supplements.** The figure provides information about the frequency of consumption of gym users who don’t use protein supplements and their weekly food intake divided in two categories: Greater than 3 times per week and 3 times or lower per week. The data are expressed as percentage.

Participants provided information of the frequency of weekly consumption of both supplements and foods. Notwithstanding the CC and the SB have broadly the same frequency of protein supplement consumption (30% and 28.8%), weekly use however differs between groups (Table 1).Male gym users demonstrated greater consumption percentages than females. The survey showed that milk is the most frequently consumed food in all groups (68% of CC and 57.8% of SB of the supplement users vs. 53% of CC and 63% of SB of non-users) followed by chicken ( 48% in CC and 50% in SB for the supplement users vs. 21% in CC and 28% in SB for non-users)(Figures 1 & 2).

Data also shows that NSU consumed significantly more snacks and bakery products than SU (P < 0.001). Interestingly, the SU consumed significantly higher quantities of vegetables, nuts, fresh fish, eggs and canned tuna (P < 0.001).

Subsequently a comparison between food categories and protein consumption was assessed (Table 2).

**Table 2:** Frequency of food intake stratified by protein content and associated with protein dietary supplements (>3 times per week)

	**Yes (%)**			**No (%)**		***p***
		**CC**	**SB**	**CC**	**SB**	
Low content (10 g or below/100 g)	Bakery	14.5	24.5	18.6	43.7	
	Milk	67.7	57.8	52.4	63.1	< 0.01
	Snack	11.3	21.6	26.2	10.7	
	Yogurt	41.9	25.5	24.8	29	
	Mean%	**33.85**	**32.35**	**29.75**	**36.6**	
Medium content (10-20 g/100 g)	Legumes	29	16.7	9	19	
	Nuts	11.3	22.5	2.8	15.9	
	Cheese	32.2	23.5	28.3	9.9	ns
	Mean%	**24.2**	**20.9**	**13.4**	**14.9**	
High content (20-25 g or above/100 g)	Meat	33.9	24.5	33.8	14.3	
	Eggs	24.1	24.5	3.4	6.3	
	Fresh Fish	22.5	7.8	10.3	4.4	< 0.01
	Chicken	48.4	50	20.7	27.8	
	Cold Cuts	29	19.6	27.6	20.6	
	Canned Tuna	22.5	23.5	6.9	9.9	
	Mean%	**30.1**	**25**	**17.1**	**13.9**	

ns. No significance.

SU eat less “low protein foods” and more “high protein foods” respect to NSU.

## Discussion

Our major interest was to understand the frequency of common foods and how this consumption varies between SU and NSU in commercial gyms. Secondly, the study focused upon the differences in consumption between the CC and SB of Palermo. Previous studies have shown discrepant rates of supplement intake amongst subjects that exercise in gyms [15,27]. These different findings might be explained by different gyms and people enrolled. Probably an under or over-reported use of such supplements, or an incorrect knowledge of what is considered a supplement may lead to such results [28,29]. Proteins are the most widely consumed supplement in commercial gyms [5,6,16], although association of protein supplements and food consumption is a poorly researched field. It is to date unclear whether those more inclined to supplement also have healthier dietary patterns. The foods that constitute the “healthy” dietary pattern are rich in vitamins, minerals and fibers, which are considered protective against non-transmissible chronic diseases [30]. These dietary patterns usually include skimmed dairy products due to low fat content.

In our study we tried to divide, at the best of our knowledge common foods, in three categories according to their protein content. Interestingly, even though no significant results occurred between our main comparison groups (CC and SB), there were significant statistical differences between those users who took supplements and those who didn’t. Participants who took supplements also ate higher protein content foods in respect to those who did not. Another noteworthy observation is the frequency consumption of bakery goods and snacks. Consumption was relatively high in both groups but significantly higher in those who didn’t use protein supplements. The data presented despite not indicating the exact amount of food ingested during each day, provided some estimate of the protein intake (INRAN database). These preliminary results seem to indicate that the participants which regularly use protein supplements have a “healthier” dietary pattern [31]. However, it‘s still uncertain if the total amount of proteins ingested is higher or lower than mean daily requirements. These results give knowledge to coaches and fitness professionals about the frequency and consumption of protein supplements. Secondly, estimation of quantity and quality of food intake of gym adepts of the city centre and the suburbs of Palermo, Italy.

## Conclusion

The results show that in resistance trained men and female gym users, the percentage of those that consume proteins is 30% in the CC and 28.8% in the SB of Palermo, Italy. Generally participants who ingest protein supplements also eat higher protein content foods. Other interesting results regard the NSU which declare a higher consumption of bakery products and snacks (foods recognized as unhealthy) respect to SU. Though, it’s not clear if the total amount of protein intake per day (g/Kg) is adequate to the physiological needs of the gym users, as the SU seem to have high protein intakes while the NSU a noticeably lower percentage. Dietary supplement industries might be interested in these research results and might invest in order to understand why this nutritional behaviour is occurring in suburban females. Further investigations are required to gain a more in-depth understanding of protein supplementation.

## Competing interests

The authors declare that they have no competing interests.

## Authors’ contributions

All authors have effectively contributed to this work in its different production stages. All authors read and approved the final manuscript.

## Supplementary Material

Additional file 1 Protein Project questionnaire adopted by Bianco et al. 2014. Click here for file
