# Supplementary material for: Protein supplementation and dietary behaviours of resistance trained men and women attending commercial gyms: a comparative study between the city centre and the suburbs of Palermo, Italy
Source: J Int Soc Sports Nutr. 2014 Jun 18;11:30. doi: 10.1186/1550-2783-11-30 (PMC4073191; doi:10.1186/1550-2783-11-30)
Supplement: Additional file 1 — Protein Project questionnaire adopted by Bianco et al. 2014. [file 1550-2783-11-30-S1.pdf]

UNIVERSITÀ DEGLI STUDI DI PALERMO

UNIVERSITÀ DEGLI STUDI DI PADOVA

proteinproject.com

Research Unit ID _______________

Date _______________

ID Participant _________________

Age (yrs) ____ Sex (m/f) ____ H (cm) ____ W (kg) ____ Education (yrs) ____

1. Do you practice any physical activities? YES ☐ NO ☐
2. If yes! What kind of activity are you practicing ? _____________________________

   2.1 Since when (years) ? _______

3. How many days per week ? _______
4. How many hours per day ? _______

Note:
____
____
____

5. Please type all places you practice physical activities __________ __________

6. Are you using protein supplements ? NO ☐ Yes ☐

   6.1 If not, have you ever used them in past ? NO ☐ Yes ☐
   (even if only once, answer yes)

   6.2 If yes, which ? (if possible, Brand / Trademark) ________________

6.2.1 Since when (months/years) ? ________________

6.2.2 How many days per week ? ________________

6.2.3 What kind (per day) ?

Protein Bar	*Whey/Egg/Mix Protein*	*Protein drink*
nr __________	gr____________	ml ______________

Mainly mixed with
Water or Milk

6.2.4 Are you taking protein supplements in association with (e.g. Creatine, Amino Acids, Weight gain, Weight loss, Vitamins, Anabolic Steroids, ecc…) ?

Please type here ____

6.2.5 Who suggested you their use:

- o Coach
- o Myself, it was my idea
- o Internet, Web
- o Physician
- o Nutritionist
- o Friends

Other: ____________________________________

7. Are you following any specific food regimen ? NO ☐ Yes ☐

If yes, please type the name here ________________________________

8. Frequency of use of the following foods:

Milk	days/week	___ _____
Cheese	days/week	___ _____
Chicken / Turkey	days/week	___ _____
Eggs	days/week	___ _____
Vegetables	days/week	___ _____
Bakery	days/week	___ _____
Cold cuts	days/week	___ _____
Yogurt	days/week	___ _____
Meat	days/week	___ _____
Fish	days/week	___ _____
Nuts	days/week	___ _____
Snacks	days/week	___ _____
Canned tuna	days/week	___ _____
Legumes	days/week	___ _____

9. Are you able to jog nonstop for 50 minutes?

- o Yes
- o No
- o I have never tried

10. Report time spent sitting during a typical day (for study purposes, work purposes or study and work purposes)

- o 1-3 hours
- o 3-5 hours
- o 5-8 hours
- o 8-10 hours
- o > 10 hours

(No weekend or holiday)
